# A Theoretical Study of the Halogen Bond between Heteronuclear Halogen and Benzene

**DOI:** 10.3390/molecules27228078

**Published:** 2022-11-21

**Authors:** Jun Luo, Hongjing Dai, Chenglu Zeng, Dawang Wu, Maoqi Cao

**Affiliations:** Qiannan Key Laboratory of Biomass-Based Functional Carbon Materials Based on Agricultural and Forestry Wastes, Qiannan Normal University for Nationalities, Duyun 558000, China

**Keywords:** halogen bond, benzene, heteronuclear halogen, interaction

## Abstract

Halogen bonds play an important role in many fields, such as biological systems, drug design and crystal engineering. In this work, the structural characteristics of the halogen bond between heteronuclear halogen XD (ClF, BrCl, IBr, ICl, BrF and IF) and benzene were studied using density functional theory. The structures of the complexes between heteronuclear halogen and benzene have Cs symmetry. The interaction energies of the complexes between heteronuclear halogen XD (ClF, BrCl, IBr, ICl, BrF and IF) and benzene range from −27.80 to −37.18 kJ/mol, increasing with the increases in the polarity between the atoms of X and D, and are proportional to the angles of a between the Z axis and the covalent bond of heteronuclear halogen. The electron density (ρ) and corresponding Laplacian (∇^2^ρ) values indicate that the interaction of the heteronuclear halogen and benzene is a typical long-range weak interaction similar to a hydrogen bond. Independent gradient model analysis suggests that the van der Waals is the main interaction between the complexes of heteronuclear halogen and benzene. Symmetry-adapted perturbation theory analysis suggests that the electrostatic interaction is the dominant part in the complexes of C_6_H_6_⋯ClF, C_6_H_6_⋯ICl, C_6_H_6_⋯BrF and C_6_H_6_⋯IF, and the dispersion interaction is the main part in the complexes of C_6_H_6_⋯BrCl, C_6_H_6_⋯IBr.

## 1. Introduction

A halogen bond is a noncovalent interaction [1,2] similar to the typical hydrogen bond [3,4,5,6]. In the case of a halogen bond, a halogen atom is shared both by a donor D and an acceptor A [7]. The halogen bond interaction can be depicted by:

D-X---A

where the X can be chlorine, bromine or iodine and the angle of D-X⋯A is close to 180°.

Halogen bonding plays an important role in many fields, such as biological systems [8,9,10,11,12], drug design [13,14,15], crystal engineering [16,17,18,19] and function materials [20,21,22,23]. Auffinger et al. explored the function of halogen bonds in ligand binding, recognition, conformational equilibria and molecular binding [24]. Johnson et al. found that the 4, 5, 6, 7-tetrabromobenzotriazole could displace charged ATP from its binding site on phospho-CDK2-cyclin by the halogen bond of Br–O interaction [25]. As a result, it is of great practical significance to study the halogen bond deeply.

Due to the influence of conjugation, right above the center of the benzene ring is an electron-rich region [26], which can combine with the halogen molecules to form stable complexes. Similar systems have been studied in recent years. Tsuzuki et al. studied the size and direction of halogen bonds of molecules containing halogen atoms interacting with benzene [27]. Schwabedissen et al. studied the action rules of halogen bonds in crystals [28]. Yu et al. studied the effect of halogen bonds on molecular fluorescence [29]. Otte et al. studied the competing weak interactions in the process of complex formation [30]. In addition, Oliveira, Kraka and Cremer et al. have published extensive work on analyzing halogen bonds based on vibrational spectroscopy, leading to a quantitative measure of the interaction strength in these systems and providing a rigorous and comprehensive discussion of halogen bonds on a quantum chemical level [31,32,33,34,35,36,37,38,39,40].

Although these studies have given the geometric structures and interaction energies of the complexes between halogen and benzene [41], to our knowledge, the details of their interaction have not been reported until now. In this work, we studied the structural characteristics of the halogen bond between heteronuclear halogen XD (ClF, BrCl, IBr, ICl, BrF and IF) and benzene using density functional theory (DFT). The calculation results indicate that heteronuclear halogen and benzene can form stable complexes with Cs symmetry. The interaction energy ranges from −27.80 to −37.18 kJ/mol, which is proportional to the angle of a. Topological properties of the electron density based on the atoms in molecules (AIM) theory show that bond critical points (BCPs) between the heteronuclear halogen and benzene exist in all the six complexes. The electron density (ρ) and corresponding Laplacian (∇^2^ρ) values indicate that the interaction of the heteronuclear halogen and benzene is a non-covalent intermolecular interaction similar to a hydrogen bond. Independent gradient model (IGM) analysis of the interaction between the heteronuclear halogen and benzene suggests that van der Waals is the main interaction.

## 2. Results and Discussion

### 2.1. Geometry and Interaction Energy

**Figure 1:**
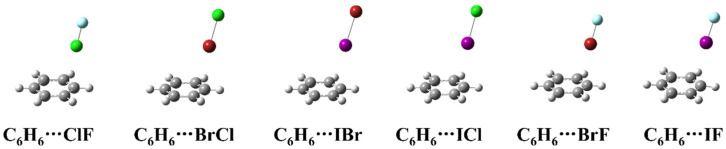
The geometric structures of the complexes between heteronuclear halogen and benzene.

Figure 1 shows the structure of the complexes formed by heteronuclear halogen (ClF, BrCl, IBr, ICl, BrF and IF) and benzene. As can be seen from the Figure 1, the heteronuclear halogen in the complexes is located above the benzene ring, but not perpendicular to the plane of the benzene ring. All the six complexes belong to the Cs symmetry, and the more electronegative atom of the heteronuclear halogen in the complexes is far away from the benzene ring.

**Figure 2:**
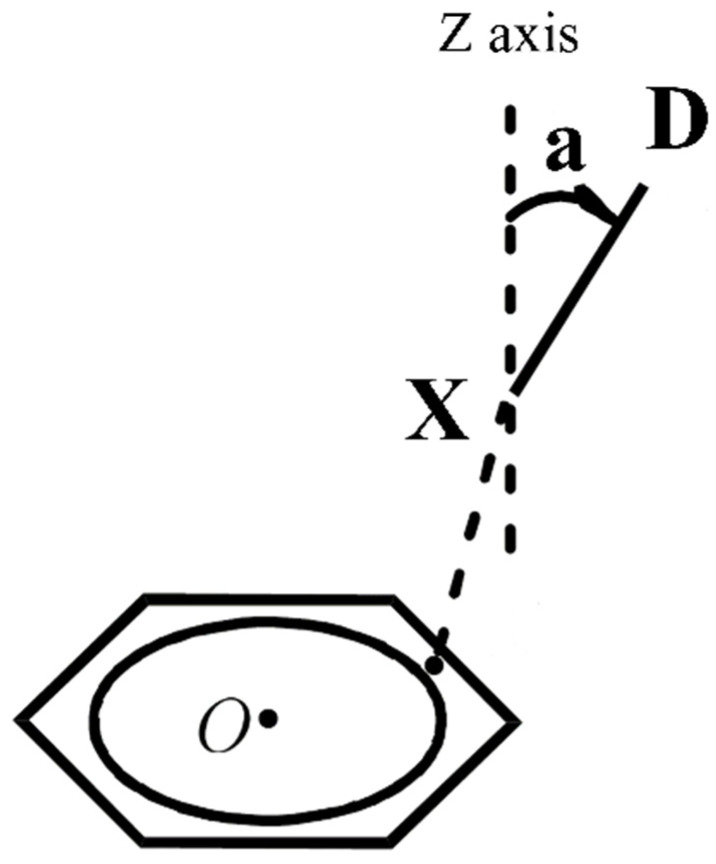
The structure diagram of the complexes between heteronuclear halogens and benzene.

Figure 2 shows the schematic diagram of the complexes. To better illustrate the relationship between the interaction energy and the structure of the complexes, the direction that passes through the X atom and is perpendicular to the plane of the benzene ring is defined as the *z*-axis, and the angle between the X−D bond and the *z*-axis is defined as a.

The values of the interaction energy and the angle of a are shown in Table 1. As can be seen from Table 1, the interaction energy is related to the magnitude of a, and increases with the increase in a. In addition, when the atom of D is fixed, the interaction energy of the complexes increases with the increase in the polarity between the atoms of X and D, which is inconsistent with the calculations from Sugibayashi, et al. [41].

**Figure 3:**
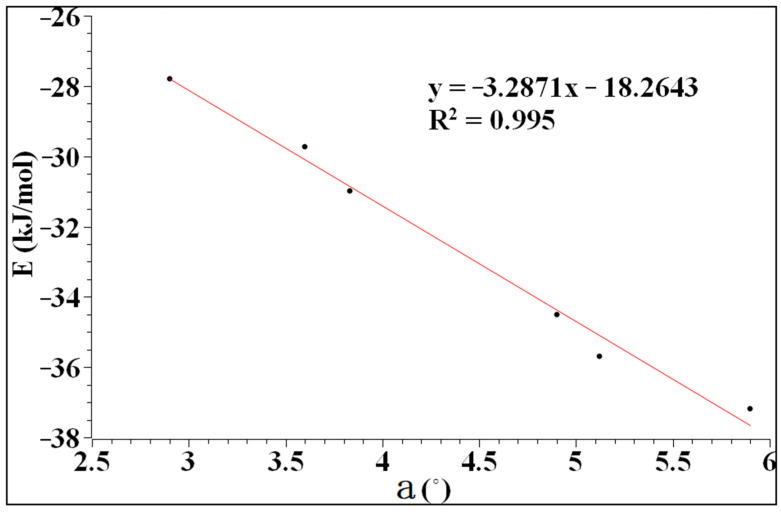
The relationship between the interaction energy and a.

As can be seen from Figure 3, the interaction energy and the angle of a have good linearity. The correlation coefficient reaches 99.5%, indicating that the interaction between heteronuclear halogen and benzene tends to be perpendicular to the plane. The interaction energy ranges from −27.80 to −37.18 kJ/mol, which is a typical long-range weak interaction similar to a hydrogen bond.

### 2.2. Electrostatic Potential

**Figure 4:**
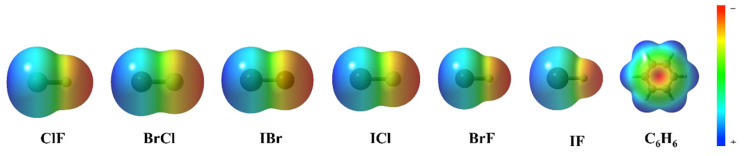
The electrostatic potential of ClF, BrCl, IBr, ICl, BrF, IF and C_6_H_6_.

Figure 4 shows the electrostatic potential of each monomer in the complexes. According to the electrostatic potential diagram, the central region of the benzene ring shows a negative electrostatic potential region. However, the heteronuclear halogen (ClF, BrCl, IBr, ICl, BrF and IF) in the complexes does not lie directly above the benzene ring but has a silent deviation from the benzene ring center. The reason for the deviation indicates that the interaction between the two monomers in the complexes is not only electrostatic. In the process of complex formation, a variety of interactions exert influence and achieve a reasonable state, resulting in complexes with Cs symmetry.

### 2.3. Topological Properties of the Electron Density

In order to further understand the interaction characteristics, we carried out an AIM theoretical analysis of the six complexes. The results show that there are bond critical points between the heteronuclear halogen and benzene in the six complexes. The values of electron density (ρ), Laplacian of electron density (∇^2^ρ) and ellipticity (ε) of the saddle point of the bond are listed in Table 2. According to the criteria proposed by Popelier et al. [42,43], the electron density (ρ) at the saddle point of the bond ranges from 0.0025 to 0.035 a.u. and the Laplacian amount of electron density (∇^2^ρ) is between 0.024 and 0.139. We found that the electron density at the saddle point of the bond and the Laplacian amount of the electron density in the six complexes are in the above range, indicating that the interaction in the complex is a weak interaction similar to that of a hydrogen bond. In addition, the ellipticity value at the saddle point of the key is related to the type of the key. As can be seen from Table 2, the ellipticity (ε) of the six complexes is all greater than zero, showing obvious π-bond characteristics.

### 2.4. IGM Analysis

**Figure 5:**
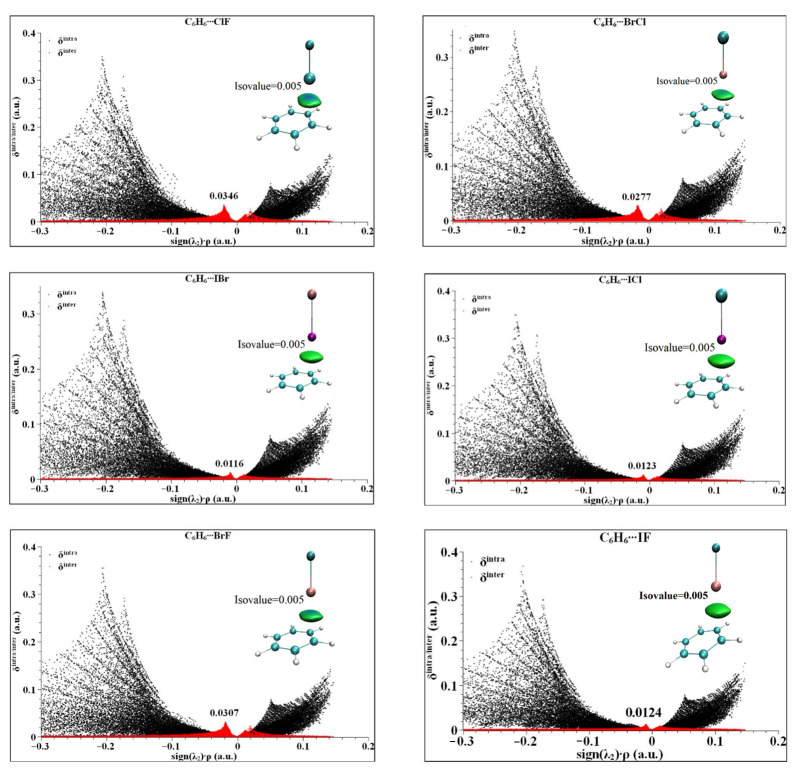
The IGM analysis and color plots of complexes between heteronuclear halogen XD (ClF, BrCl, IBr, ICl, BrF and IF) and benzene.

In order to understand the region, size and species of the weak interaction of the halogen bond between the heteronuclear halogen and benzene in the six complexes, IGM analysis of the six complexes was carried out [44,45]. In IGM theory, the second largest eigenvalue of the electron density Hessian matrix (sign (λ_2_)) is used to define the type of interaction [46]. When the value of sign (λ_2_) × ρ is less than zero, it is an attractive interaction. When the value of sign (λ_2_) × ρ is greater than zero, it is a repulsive interaction. When the value of sign (λ_2_) × ρ is equal to zero, it is a van der Waals interaction. IGM is calculated by distinguishing the interaction into intramolecular and intermolecular interactions, which are represented by δ^inter^ and δ^intra^, respectively. The peak value of δ^inter^ is generally less than 0.1 a.u., the van der Waals interaction is generally less than 0.03 a.u., and the hydrogen bond interaction is generally less than 0.1 a.u. The calculated results of the six complexes are shown in Figure 5. As can be seen from Figure 5, all six complexes have different degrees of peak values in the region of sign (λ_2_) × ρ < 0, indicating that the interactions in the complexes are all in the van der Waals intermolecular interaction region. The peak values of δ^inter^ are from 0.0125 to 0.0476 a.u., where C_6_H_6_⋯BrF and C_6_H_6_⋯ClF exceed 0.003 a.u., indicating that the complexes exhibit a strong van der Waals interaction, and the remaining C_6_H_6_⋯BrCl, C_6_H_6_⋯ICl, C_6_H_6_⋯IBr and C_6_H_6_⋯IF shows a general van der Waals effect. From the isosurface color map in Figure 5, the color map of the six complexes shows that the majority of the results are green, which further indicates that the van der Waals is the dominant interaction. At the same time, it can be seen that the central region of the isosurface is blue, indicating that the strongest interaction position is between the X atom and the C-C bond center, which is consistent with the above conclusion of electron density topological analysis.

### 2.5. SAPT Calculation Analysis

In order to further understand the tendency and characteristics of the van der Waals interaction between heteronuclear halogen and benzene, the intermolecular interaction energy was decomposed and calculated using symmetry-adapted perturbation theory (SAPT). The calculation results are listed in Table 3. In the six complexes, the electrostatic and dispersive energies are both larger than the induced energies, indicating that the electrostatic and dispersive effects are dominant in the six complexes. Further studies reveal that in C_6_H_6_⋯ClF, C_6_H_6_⋯ICl, C_6_H_6_⋯BrF and C_6_H_6_⋯IF, electrostatic energy accounts for the largest proportions, which are 36.7%, 37.3%, 37.9% and 38.9%, respectively, indicating that the electrostatic interaction of these four complexes is dominant. In C_6_H_6_⋯BrCl and C_6_H_6_⋯IBr, the dispersive energy has the largest proportions of the interaction energy, which are 37.9% and 38.6%, respectively, indicating that the dispersive interaction in the two complexes is the main part. The dispersion effect is more obvious in the complexes with large radius halogen atoms, which might be related to the degree of electron dispersion in halogen atoms.

**Figure 6:**
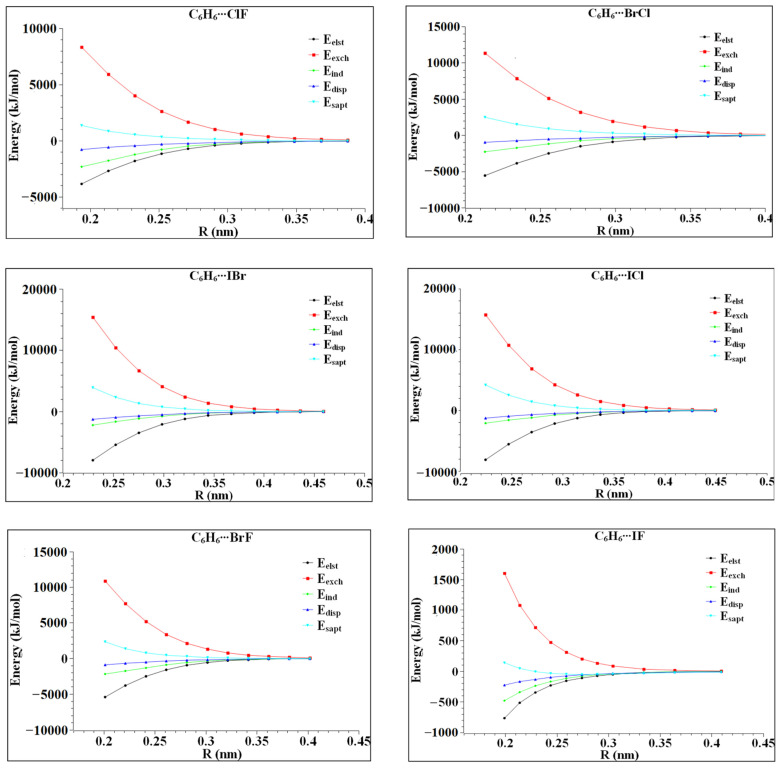
The interaction energy varies with the distance from the centroid of heteronuclear halogen XD.

In order to further study the role of the interaction energies, such as induction, dispersion, exchange and electrostatic energy in complex formation, the variation of each energy with the distance of its monomer centroid was calculated. The calculation results are shown in Figure 6. It can be seen in Figure 6 that the variation in each interaction energy with the distance from the centroid of the monomer is not completely the same, but the variation trend is similar. In the range of R < 0.3 nm, the exchange and induction effects are significantly affected by the molecular spacing, and the repulsive and attractive effects are basically equal. At this time, the intermolecular interaction shows electrostatic interaction and dispersion interaction, but the dispersion interaction line is always below the electrostatic interaction line, indicating that the dispersion interaction is obviously affected. At R = 0.4 nm, the six complexes reach equilibrium. Finally, C_6_H_6_⋯ClF, C_6_H_6_⋯ICl, C_6_H_6_⋯BrF and C_6_H_6_⋯IF are dominated by electrostatic interaction, while the dispersion interaction in C_6_H_6_⋯BrCl, C_6_H_6_⋯IBr is dominant. Among the six complexes, the dispersion interaction line is the flattest, indicating that the intermolecular distance is the least affected. In C_6_H_6_⋯ICl and C_6_H_6_⋯IBr, the electrostatic action line and the dispersion action line are very close, indicating that the electrostatic action and dispersion action are similar.

## 3. Materials and Methods

The geometric structures of heteronuclear halogen, benzene and their complexes were optimized using DFT with the functional of wB97XD and the basis set of aug-cc-pVTZ. The calculations for iodine were carried out with quasi-relativistic small-core effective potentials (ECPs) and the corresponding Peterson AVTZ basis set. The frequency calculation was conducted with the same base set as structure optimization and the results show that heteronuclear halogen, benzene and their complexes have no imaginary frequency. The interaction energy was calculated with the same level of theory as the geometry optimization. The counterpoise procedure of Boys et al. was used for BSSE correction of the base group overlap errors [47]. The program used for calculation was Gaussian 16 [48]. The AIM theory was used for electron density topological analysis and electrostatic potential analysis on the basis of wave function from Gaussian in order to further discuss the characteristics of the interaction between heteronuclear halogens and benzene [49,50]. SAPT was used to study the composition and interaction of molecular interaction energy with the same base set as structure optimization [51,52]. The calculation program was Psi4 [53].

## 4. Conclusions

Heteronuclear halogen and benzene can form stable complexes with Cs symmetry. The interaction energy is from −27.80 to −37.18 kJ/mol. All six complexes show obvious hydrogen bond-like properties with a weak long-range interaction. The van der Waals is the main interaction between the complexes of heteronuclear halogen and benzene. The electrostatic interaction is the dominant part in the complexes of C_6_H_6_⋯ClF, C_6_H_6_⋯ICl, C_6_H_6_⋯BrF and C_6_H_6_⋯IF, and the dispersion interaction is main part in the complexes of C_6_H_6_⋯BrCl, C_6_H_6_⋯IBr.

## Figures and Tables

**Table 1:** The values of the interaction energy and the angle of a.

Complexes	a (°)	(E) Interaction Energy (kJ/mol)
C_6_H_6_⋯ClF	2.90	−27.80
C_6_H_6_⋯BrCl	3.60	−29.72
C_6_H_6_⋯IBr	3.83	−30.99
C_6_H_6_⋯ICl	4.90	−34.50
C_6_H_6_⋯BrF	5.12	−35.70
C_6_H_6_⋯IF	5.56	−37.18

**Table 2:** The values of electron density (ρ), Laplacian of electron density (▽^2^ρ) ellipticity (ε) and λ_2_ of the saddle point.

Complex	ρ	∇^2^ρ	ε	λ_2_
C_6_H_6_⋯ClF	0.019	0.059	4.39	−0.0026
C_6_H_6_⋯BrCl	0.015	0.049	3.80	−0.0019
C_6_H_6_⋯IBr	0.014	0.024	4.00	−0.0017
C_6_H_6_⋯ICl	0.015	0.039	4.41	−0.0016
C_6_H_6_⋯BrF	0.019	0.054	4.50	−0.0021
C_6_H_6_⋯IF	0.019	0.061	4.76	−0.0023

**Table 3:** The calculation analysis using symmetry matching perturbation theory (SAPT).

Energy (kJ/mol)	C_6_H_6_⋯ClF	C_6_H_6_⋯BrCl	C_6_H_6_⋯IBr	C_6_H_6_⋯ICl	C_6_H_6_⋯BrF	C_6_H_6_⋯IF
E_SAPT_	−27.86	−29.70	−31.04	−34.57	−35.79	−37.33
E_exch_	35.95	43.35	44.83	46.37	42.84	47.12
E_elst_	−23.46	−26.29	−27.74	−30.22	−29.85	−34.04
	(36.7%)	(35.9%)	(36.6%)	(37.3%)	(37.9%)	(38.9%)
E_ind_	−18.15	−19.03	−18.84	−21.26	−23.04	−25.39
	(28.4%)	(26.1%)	(24.8%)	(26.3%)	(29.3%)	(29.0%)
E_disp_	−22.20	−27.73	−29.29	−29.45	−25.74	−28.02
	(34.8%)	(37.9%)	(38.6%)	(36.4%)	(32.7%)	(32.0%)

## Data Availability

Not applicable.

## Sample Availability

Samples of the compounds are not available from the authors.
